# The safety of nintedanib for the treatment of interstitial lung disease: A systematic review and meta-analysis of randomized controlled trials

**DOI:** 10.1371/journal.pone.0251636

**Published:** 2021-05-14

**Authors:** Chao-Hsien Chen, Hui-Chuan Lin, Ya-Hui Wang, Cheng-Yi Wang, You Shuei Lin, Chih-Cheng Lai

**Affiliations:** 1 Division of Pulmonary, Department of Internal Medicine, MacKay Memorial Hospital, Taipei, Taiwan; 2 Department of Medicine, MacKey Medical College, New Taipei City, Taiwan; 3 Division of Respiratory Therapy, Department of Chest Medicine, Cardinal Tien Hospital, New Taipei City, Taiwan; 4 Medical Research Center, Cardinal Tien Hospital and School of Medicine, College of Medicine, Fu Jen Catholic University, New Taipei City, Taiwan; 5 Department of Internal Medicine, Cardinal Tien Hospital and School of Medicine, College of Medicine, Fu Jen Catholic University, New Taipei City, Taiwan; 6 Department of Physiology, School of Medicine, College of Medicine, Taipei Medical University, Taipei, Taiwan; 7 Department of Internal Medicine, Kaohsiung Veterans General Hospital, Tainan Branch, Tainan, Taiwan; University of Texas McGowan Medical School at Houston, UNITED STATES

## Abstract

**Introduction:**

Nintedanib can inhibit processes involved in the progression of fibrosis and can reduce the decline in forced vital capacity in patients with idiopathic pulmonary fibrosis (IPF) and fibrotic-interstitial lung disease (fibrotic-ILDs). Although the adverse events associated with nintedanib in IPF patients are well known, its safety in other fibrotic-ILD patients remained unclear.

**Methods:**

We searched PubMed, EMBASE, Cochrane CENTRAL and Cochrane CDSR for randomized controlled studies which compared nintedanib with a placebo in ILD patients. We estimated pooled odds ratios (ORs) and 95% confidence intervals (CIs) for adverse events using the DerSimonian–Laird random-effects model.

**Results:**

Six studies with a total of 2,583 patients were included in the meta-analysis. The pooled estimates showed that patients treated with nintedanib had a significantly higher likelihood of having any adverse events (OR = 2.39; 95% CI = 1.71–3.36) or adverse events leading to treatment discontinuation (OR = 1.73; 95% CI = 1.34–2.25). However, they had trend to lower likelihood of having fatal adverse events (OR = 0.69; 95% CI = 0.41–1.14) compared with the placebo group. Use of nintedanib was positively associated with diarrhea (OR = 5.96; 95% CI = 4.35–8.16), nausea (OR = 3.00; 95% CI = 1.93–4.66), vomiting (OR = 3.22; 95% CI = 2.17–4.76) and weight loss (OR = 3.38; 95% CI = 1.1.76–6.47). Whereas, patients treated with nintedanib were less likely to have a cough (OR = 0.73; 95% CI = 0.56–0.96) and dyspnea (OR = 0.70; 95% CI = 0.53–0.94).

**Conclusions:**

Compared to a placebo, nintedanib was associated with a higher risk of adverse events, especially for diarrhea, nausea, vomiting and weight loss, but it was also associated with a lower risk of cough and dyspnea in IPF and fibrotic-ILD patients.

## Introduction

Interstitial lung disease (ILD) is a group of lung diseases affecting the interstitium, which can result in restrictive lung defects and impaired gas-exchange. Idiopathic pulmonary fibrosis (IPF) is the most common of the idiopathic interstitial pneumonias and most severe form of ILD, and is characterized by progressive fibrosis of the lung parenchyma occurring primarily in older adults due to an unknown cause. The prognosis for patients with IPF is quite poor with a median survival time of 2 to 3 years if left untreated; the disease has a variable clinical course [1, 2]. There are other forms of ILD which can present with progressive fibrosis, including connective tissue disease-related ILDs, ILD related to chronic sarcoidosis, chronic hypersensitivity pneumonitis, idiopathic non-specific interstitial pneumonia and unclassifiable ILD. Patients with these fibrotic-ILDs have early mortality and are believed to have similar underlying pathogenetic mechanisms to IPF [3].

Nintedanib is a tyrosine-kinase inhibitor that mainly targets platelet-derived growth factor receptor, vascular endothelial growth factor receptor, and fibroblast growth factor receptor [4]. It can inhibit processes involved in fibrosis progression [5]. Previous studies have suggested that nintedanib could reduce the decline in forced vital capacity (FVC), preserve quality of life, lower the incidence of acute exacerbations, and increase survival time in patients with IPF [6–8]. In previous network meta-analysis of randomized controlled trials (RCTs) comparing 11 treatments in IPF, nintedanib was 1 of 4 medications had benefit, including in pulmonary function decline, exacerbation and mortality [9–13]. More recent studies have shown that nintedanib can also reduce the decline in FVC in patients with systemic sclerosis (SSc)–associated ILD and progressive fibrosis ILD (PF-ILD) in addition to those with IPF [14, 15].

Although early initiation of anti-fibrotic treatment to preserve health lung tissue is recommended [16], the possible side effects of the drugs, the symptoms of the lung disease and comorbidities due to old age can make this decision more complicated. A real world retrospective observational study of 224 IPF cases treated with nintedanib revealed that 55.7% of patients had adverse events, 28.3% of patients received a reduced treatment dose, and 13.1% of patients had to discontinue nintedanib [17]. Adverse events are the main reason for early discontinuing in clinical practice.

Cumulative evidence has focused on the safety and tolerability of nintedanib in IPF patients by evaluating a wide range of data, including clinical trials, post-hoc analyses of clinical trials, post-marketing surveillance, and real-world or epidemiological data [18–20]. However, the safety profile of nintedanib for fibrotic-ILD patients remains unclear. In a recent network meta-analysis, nintedanib was one of three medications for SSc-associated ILD resulting higher withdrawing due to adverse events compared with placebo [21]. Therefore, we performed a comprehensive systematic review and meta-analysis of double-blinded, RCTs in patients with IPF and other forms of fibrotic-ILDs to evaluate adverse events when they were treated with nintedanib compared with placebo.

## Materials and methods

### Search strategy

A literature search was performed according to Preferred Reporting Items for Systematic Reviews and Meta-Analyses guidelines [22]. PubMed, Embase, Cochrane Central Trials databases and the Cochrane Database of Systematic Reviews (CDSR) were searched for prospective, double blinded, RCTs published from inception to 29^th^ January 2020. The text and medical subject heading (MeSH) terms included: "nintedanib" [MeSH term], Ofev, Vargatef, BIBF 1120, BIBF1120, and BIBF-1120 [Text Word]. Articles were not limited to the English language. Reference lists were also searched for additional eligible articles.

### Study selection and data extraction

Two investigators (Chen and Wang) independently screened and reviewed each study. Studies were included if they met the following criteria: (1) patients with ILD, (2) prospective, double blinded RCT, (3) nintedanib as the intervention, (4) placebo as the comparison, (5) a study outcome of adverse events. Populations with any malignancies were excluded.

The following information was extracted from the included studies: the name of the trial, year of publication, intervention groups, patient number, duration of trial, randomization procedures, study population, age, sex, and interval since diagnosis with IPF. Data extraction was performed by two independent reviewers. A third reviewer (Lin) was consulted to resolve any disagreements.

### Quality assessment

The quality of each included study was assessed using a risk-of-bias assessment tool [23]. Two reviewers subjectively reviewed all included studies and rated then “low risk,” “high risk,” or “unclear” according to the following items: randomization sequence generation, allocation concealment, blinding of participants and personnel, blinding of outcome assessment, incomplete outcome data, selective reporting, and inclusion of intention-to-treat analyses. Any disagreement was resolved and decided by a third reviewer.

### Outcome measures and statistical analysis

The primary outcome of this study was adverse events, including severe, serious and fatal adverse events. In addition, the most common adverse events were listed and included in the analyses. The odds ratio (OR) and 95% confidence interval (CI) were used as the measure of association between adverse events and the use of nintedanib.

A DerSimonian–Laird random-effects model was performed to calculate the pooled estimates of ORs [24]. A two-sided P value of <0.05 was considered to indicate a significant difference. Study heterogeneity was presented using a χ^2^-based Cochran’s Q statistic and I^2^. Cochran’s Q was defined by summing the square of the amount that each study’s estimate deviated from the overall estimate. For the Q statistic, P values <0.10 were considered statistically significant for heterogeneity. For the I^2^ statistic, heterogeneity was assessed as follows: no heterogeneity (I^2^ = 0–25%), moderate heterogeneity (I^2^ = 25–50%), large heterogeneity (I^2^ = 50–75%), and extreme heterogeneity (I^2^ = 75–100%). A sensitivity analysis was conducted using a leave-one-out approach. All statistical analyses were performed using Review Manager version 5.3.

Adverse events were defined according to the FDA and previous studies, including TOMORROW [6], INPULSIS [7], NCT01979952 [25], INBUILD [15] and SENSCIS [14]. Safety was assessed by means of clinical and laboratory evaluation at study visits and recording of adverse events. In the studies, the frequency and severity of adverse events were documented according to the Medical Dictionary for Regulatory Activities, version 16.1. An adverse event was defined as any untoward medical occurrence associated with the use of the drug in humans, whether or not it was considered drug related. Severe adverse events were defined as events that were incapacitating or that caused the inability to work or to perform usual activities. Serious adverse events were defined as an event that resulted in death, in hospitalization or the prolongation of hospitalization, or a persistent or clinically significant disability or incapacity; or was life-threatening, or a congenital anomaly or birth defect, or deemed to be serious for any other reason. Fatal adverse events were defined as death caused by treatment.

## Results

### Literature search and evaluation for study inclusion

**Fig 1:**
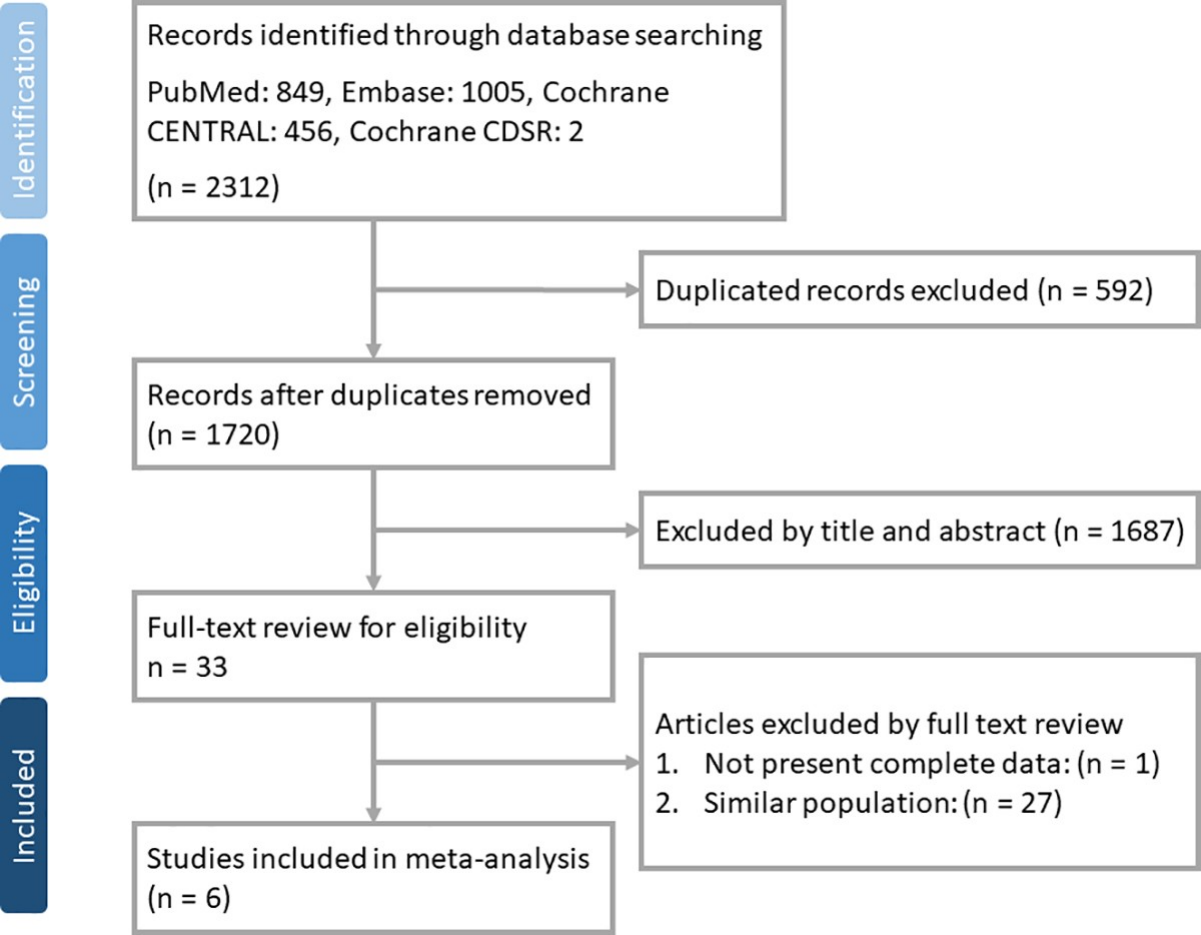
Flow chart of study selection.

A total of 2,312 articles were identified from a search of PubMed (n = 849), EMBASE (n = 1,005), Cochrane CENTRAL (n = 456), and Cochrane CDSR (n = 2). After removing duplicate records (n = 592) and ineligible articles based on a review of their title and abstract (n = 1,687) a total of 33 studies remained. A further 28 articles were removed after a full-text review process, so a total of 6 studies were included in the final study (Fig 1).

### Study characteristics

Table 1 shows the characteristics of the 6 included trials from four different articles [6, 7, 14, 15] and one unpublished trial [25]. A total of 2,583 patients were included in the meta-analysis (1,399 in the nintedanib group and 1,184 in the placebo group). One trial (NCT01979952) [25] was completed in 2016 but has not been published. That study did not describe the randomization procedures in detail and the duration of the trial was only 6 months, which was shorter than the other included trials. Nintedanib was administered twice daily with a dosage of 150 mg in five trials [6, 7, 14, 15]. The TOMORROW study included four nintedanib groups with different dosages (50 mg once daily, 50 mg twice daily, 100 mg twice daily, and 150 mg twice daily) [6]. Stratified randomization was performed in two studies [14, 15]. The INBUILD study stratified patients according to UIP-like fibrotic patterns, while the SENSCIS study stratified patients based on the presence of anti-topoisomerase I antibody. Two studies [14, 15] recruited patients ≥18 years of age while others had patients aged ≥40 years. The SENSCIS study enrolled patients who were younger (54.6±11.8 years in the nintedanib group and 53.4±12.6 in the placebo group) and had a higher proportion of female patients compared with the other studies. The mean interval since diagnosis of IPF ranged from 1.0 to 1.7 years, as reported by 3 studies [6, 7] (Table 1). In total, >90% of patients had adverse events during the study period, especially in the nintedanib group. Five trials with a follow-up duration of 52 weeks were included. In the TOMORROW study, only patients treated with nintedanib 150 mg twice daily and patients in the placebo group were included for comparison. Details of the studies are described in Table 2.

**Table 1:** Summary of baseline characteristics of included studies.

Trial name	Year of publication	Intervention	Patient number	Duration of trial^a^	Randomization	Study population	Age (yr)^b^	Male (%)	Interval since diagnosis of IPF (yr)^b^
INBUILD [15]	2019	Nintedanib 150 mg bid	332	52 weeks	1:1 ratio with interactive-response technology and stratified according to UIP-like fibrotic pattern or not. An enrichment design was performed to ensure two thirds of patients having UIP-like pattern.	At least 18 y/o with fibrosing interstitial lung disease and with a more progressive fibrotic phenotype. Having features of fibrosing lung disease affecting more than 10% of lung volume on HRCT. FVC≧ 45% of predicted value and DL_CO_ between 30% and 80% of predicted value.	65.2±9.7	53.9	NR
		Placebo	331				66.3±9.8	53.5	NR
INPULSIS-1 [7]	2014	Nintedanib 150 mg bid	309	52 weeks	3:2 ratio by an interactive telephone and web-based response system.	At least 40 y/o with diagnosis of IPF within 5 years and had an FVC≧50% of predicted value, and had DL_CO_ between 30% and 79%.	66.9±8.4	81.2	1.7±1.4
		Placebo	204				66.9±8.2	79.9	1.6±1.4
INPULSIS-2 [7]	2014	Nintedanib 150 mg bid	329	52 weeks	The same as above.	The same as above.	66.4±7.9	77.8	1.6±1.3
		Placebo	219				67.1±7.5	78.1	1.6±1.3
NCT01979952 [25]	2016^c^	Nintedanib 150 mg bid	56	6 months (up to 18 months)	NR	At least 40 y/o with IPF diagnosis confirmed by HRCT and had an FVC≧50% of predicted value, and had DL_CO_ between 30% and 79%.	NR	80.4	NR
		Placebo	57				NR	64.9	NR
SENSCIS [14]	2019	Nintedanib 150 mg bid	288	52 weeks	1:1 ratio with an interactive response system and stratified by the presence of antitopoisonmerase I antibody.	At least 18 y/o and had systemic sclerosis with the first onset of non-Raynaud’s symptom within 7 years, had an FVC≧40% of predicted value, and had DL_CO_ between 30% and 89% of predicted value. ILD was defined as fibrosis affecting at least 10% of lungs by HRCT.	54.6±11.8	23.3	NR
		Placebo	288				53.4±12.6	26.4	NR
TOMORROW [6]	2011	Nintedanib 50 mg qd	86	52 weeks	Patients were randomized into four treatment groups and placebo group. A stepwise increasing-dose approach was used.	At least 40 y/o with IPF and had a FVC≧50% of predicted value, had DL_CO_ between 30% and 79% of predicted value, and had a partial PaO_2_ either when breathing ambient air≧55 mmHg or greater at altitudes up to 1500 m.	65.3±9.4	75.6	1.4±1.3
		Nintedanib 50 mg bid	86				64.9±8.5	72.1	1.1±1.2
		Nintedanib 100 mg bid	86				65.1±8.6	75.6	1.2±1.2
		Nintedanib 150 mg bid	85				65.4±7.8	76.5	1.0±1.2
		Placebo	85				64.8±8.6	74.1	1.4±1.5

IPF, idiopathic pulmonary fibrosis; FVC, forced vital capacity; DL_CO_, diffusing capacity of the lung for carbon monoxide; SpO_2_, oxygen saturation of peripheral blood; NR, not reported.

^a^Defined by the duration from start of trial to the time of end points measurements.

^b^Presented as mean±standard deviation.

**Table 2:** Summary of adverse events of five published trials^a^.

		No. of patients (%)											
		INBUILD		INPULSIS-1		INPULSIS-2		SENSCIS		TOMORROW^b^		Total	
Events		Nintedanib	placebo	Nintedanib	placebo	Nintedanib	placebo	Nintedanib	placebo	Nintedanib	placebo	Nintedanib	placebo
		(N = 332)	(N = 331)	(N = 309)	(N = 204)	(N = 329)	(N = 219)	(N = 288)	(N = 288)	(N = 85)	(N = 85)	(N = 1343)	(N = 1127)
Any adverse event		317 (95.5)	296 (89.4)	298 (96.4)	181 (88.7)	311 (94.5)	198 (90.4)	283 (98.3)	276 (95.8)	80 (94.1)	77 (90.6)	1289 (96.0)	1031 (91.5)
Severe adverse event		60 (18.1)	73 (22.1)	81 (26.2)	37 (18.1)	93 (28.3)	62 (28.3)	52 (18.1)	36 (12.5)	19 (22.4)	20 (23.5)	305 (22.7)	228 (20.2)
Serious adverse event		107 (32.2)	110 (33.2)	96 (31.1)	55 (27.0)	98 (29.8)	72 (32.9)	69 (24.0)	62 (21.5)	23 (27.1)	26 (30.6)	393 (29.3)	325 (28.8)
Fatal adverse event		11 (3.3)	17 (5.1)	12 (3.9)	10 (4.9)	25 (7.6)	21 (9.6)	5 (1.7)	4 (1.4)	1 (1.2)	12 (14.1)	54 (4.0)	64 (5.7)
Most frequent adverse event													
	Diarrhea	222 (66.9)	79 (23.9)	190 (61.5)	38 (18.6)	208 (63.2)	40 (18.3)	218 (75.7)	91 (31.6)	47 (55.3)	13 (15.3)	885 (65.9)	261 (23.2)
	Nausea	96 (28.9)	31 (9.4)	70 (22.7)	12 (5.9)	86 (26.1)	16 (7.3)	91 (31.6)	39 (13.5)	20 (23.5)	8 (9.4)	363 (27.0)	106 (9.4)
	Vomiting	61 (18.4)	17 (5.1)	40 (12.9)	4 (2.0)	34 (10.3)	7 (3.2)	71 (24.7)	30 (10.4)	11 (12.9)	4 (4.7)	217 (16.2)	62 (5.5)
	Nasopharyngitis	44 (13.3)	40 (12.1)	39 (12.6)	34 (16.7)	48 (14.6)	34 (15.5)	36 (12.5)	49 (17.0)	6 (7.1)	11 (12.9)	173 (12.9)	168 (14.9)
	Cough	33 (9.9)	44 (13.3)	47 (15.2)	26 (12.7)	38 (11.6)	31 (14.2)	34 (11.8)	52 (18.1)	8 (9.4)	17 (20.0)	160 (11.9)	170 (15.1)
	Weight loss	41 (12.3)	11 (3.3)	25 (8.1)	13 (6.4)	37 (11.2)	2 (0.9)	34 (11.8)	12 (4.2)			137 (10.2)	38 (3.4)
	Decrease appetite	48 (14.5)	17 (5.1)	26 (8.4)	14 (6.9)	42 (12.8)	10 (4.6)			13 (15.3)	0 (0.0)	129 (9.6)	41 (3.6)
	Bronchitis	41 (12.3)	47 (14.2)	36 (11.7)	28 (13.7)	31 (9.4)	17 (7.8)			9 (10.6)	11 (12.9)	117 (8.7)	103 (9.1)
	Upper respiratory tract infection			28 (9.1)	18 (8.8)	30 (9.1)	24 (11.0)	33 (11.5)	35 (12.2)	7 (8.2)	13 (15.3)	98 (7.3)	90 (8.0)
	Dyspnea	36 (10.8)	44 (13.3)	22 (7.1)	23 (11.3)	27 (8.2)	25 (11.4)			6 (7.1)	11 (12.9)	91 (6.8)	103 (9.1)
	Progression of IPF (ILD)	16 (4.8)	39 (11.8)	31 (10.0)	21 (10.3)	33 (10.0)	40 (18.3)			4 (4.7)	11 (12.9)	84 (6.3)	111 (9.8)
	Abdominal pain	34 (10.2)	8 (2.4)					33 (11.5)	21 (7.3)	10 (11.8)	3 (3.5)	77 (5.7)	32 (2.8)
	skin ulcer							53 (18.4)	50 (17.4)			53 (3.9)	50 (4.4)
	Headache	35 (10.5)	23 (6.9)							11 (12.9)	5 (5.9)	46 (3.4)	28 (2.5)
	Alanine aminotransferase increased	43 (13.0)	12 (3.6)									43 (3.2)	12 (1.1)
	Fatigue							31 (10.8)	20 (6.9)	9 (10.6)	7 (8.2)	40 (3.0)	27 (2.4)
	Aspartate aminotransferase increased	38 (11.4)	12 (3.6)									38 (2.8)	12 (1.1)
Adverse events leading to discontinuation		65 (19.6)	34 (10.3)	65 (21.0)	22 (10.8)	58 (17.6)	33 (15.1)	46 (16.0)	25 (8.7)	26 (30.6)	22 (25.9)	259 (19.3)	136 (12.1)
	GI disorder			26 (8.4)	3 (1.5)	21 (6.4)	2 (0.9)			14 (16.5)	2 (2.4)	61 (4.5)	7 (0.6)
	Respiratory, thoracic & mediastonal disorder			12 (3.9)	10 (4.9)	8 (2.4)	18 (8.2)			4 (4.7)	10 (11.8)	24 (1.8)	38 (3.4)
	Investigation results			10 (3.2)	1 (0.5)	8 (2.4)	1 (0.5)					18 (1.3)	2 (0.2)
	Cardiac disorder			5 (1.6)	4 (2.0)	2 (0.6)	3 (1.4)			0 (0.0)	6 (7.1)	7 (0.5)	13 (1.2)
	General disorder and condition involving site of study-drug administration			8 (2.6)	3 (1.5)	2 (0.6)	1 (0.5)					10 (0.7)	4 (0.4)
	Infections and infestations									0 (0.0)	6 (7.1)	0 (0.0)	6 (0.5)

^a^NCT01979952 is excluded due to different follow-up duration from other included trials.

^b^Only patients treated with nintedanib 150 mg twice daily and placebo were selected for comparison.

### Meta-analyses for the association between adverse events and the use of nintedanib

**Fig 2:**
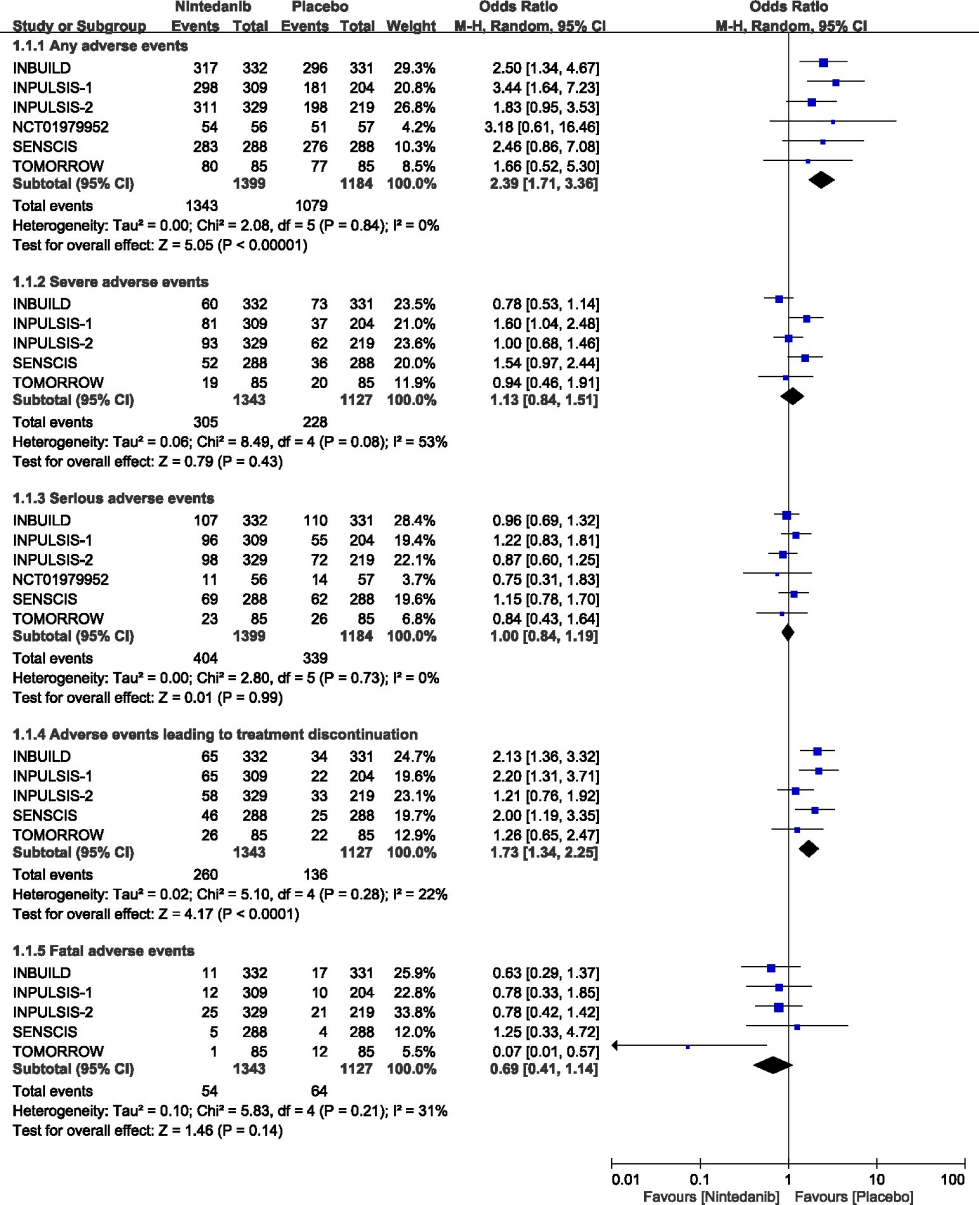
Forest plots for association between adverse events and use of nintedanib. NCT01979952 was not showed up in 1.1.2, 1.1.4 and 1.1.5 for no results available.

Fig 2 shows forest plots of the meta-analysis for the six included studies [6, 7, 14, 15, 25]. There was no heterogeneity across the six studies for any adverse events (Q = 2.08; P = 0.84; I^2^ = 0%), serious adverse events (Q = 2.80; P = 0.73; I^2^ = 0%), or adverse events leading to treatment discontinuation (Q = 5.10; P = 0.28; I^2^ = 22%). However, there was moderate heterogeneity for fatal adverse events (Q = 5.83; P = 0.21; I^2^ = 31%) and high heterogeneity for severe adverse events (Q = 8.49; P = 0.08; I^2^ = 53%).

Pooling estimates showed that patients treated with nintedanib had a significantly higher likelihood of having any adverse events (OR = 2.39; 95% CI = 1.71–3.36; P < 0.001) and having adverse events leading to treatment discontinuation (OR = 1.73; 95% CI = 1.34–2.25; P < 0.001). Whereas, they had a trend to lower likelihood of having a fatal adverse event (OR = 0.69; 95% CI = 0.41–1.14; P = 0.14) compared with the placebo group.

**Fig 3:**
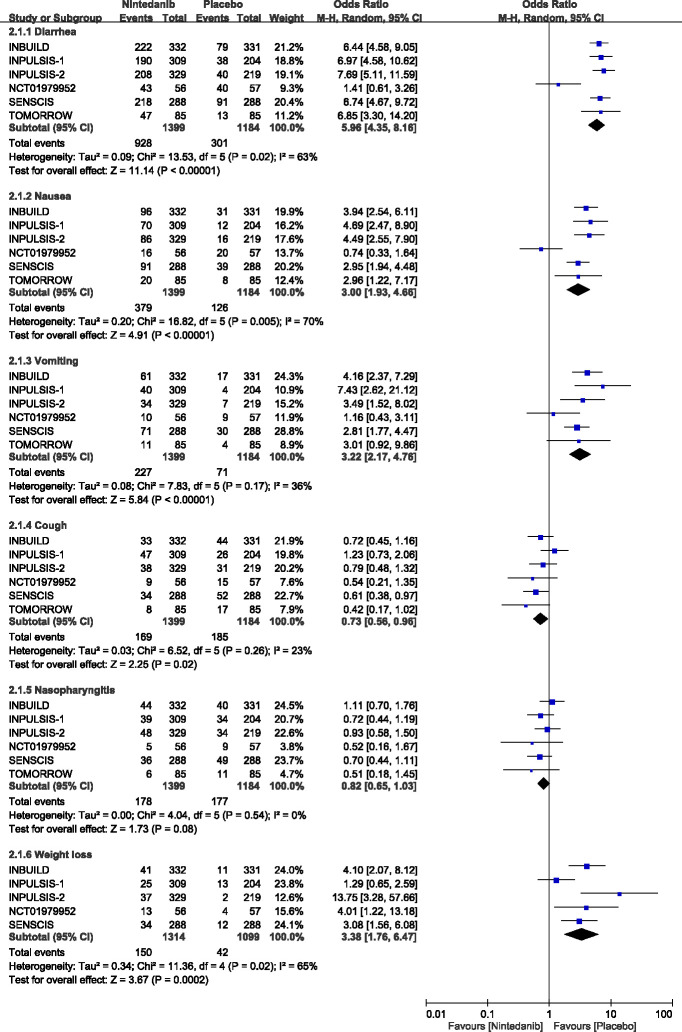
Forest plots for association between the most frequent adverse events and use of nintedanib.

Fig 3 and S1 Fig shows the pooled results for the most frequent adverse events. There was a large heterogeneity observed for diarrhea (Q = 13.53; P = 0.02; I^2^ = 63%), nausea (Q = 16.82; P = 0.005; I^2^ = 70%), weight loss (Q = 11.36; P = 0.02; I^2^ = 65%), abdominal pain (Q = 8.02; P = 0.05; I^2^ = 63%), and decreased appetite (Q = 8.27; P = 0.08; I^2^ = 52%). Moderate heterogeneity existed among the 6 studies for vomiting (Q = 7.83; P = 0.17; I^2^ = 36%), while no heterogeneity was observed for cough (Q = 6.52; P = 0.26; I^2^ = 23%), nasopharyngitis (Q = 4.04; P = 0.54; I^2^ = 0%), bronchitis (Q = 1.78; P = 0.78; I^2^ = 0%), dyspnea (Q = 1.05; P = 0.90; I^2^ = 0%), and upper respiratory tract infection (Q = 2.13; P = 0.71; I^2^ = 0%).

Use of nintedanib was associated with the higher risk of diarrhea (66.3% vs 25.4%; OR = 5.96; 95% CI = 4.35–8.16; P < 0.001), nausea (27.1% vs 10.6%; OR = 3.00; 95% CI = 1.93–4.66; P < 0.001), vomiting (16.2% vs 6.0%; OR = 3.22; 95% CI = 2.17–4.76; P < 0.001), weight loss (11.4% vs 3.8%; OR = 3.38; 95% CI = 1.76–6.47; P < 0.001),and decreased appetite (12.7% vs 5.2%; OR = 2.53; 95% CI = 1.45–4.39; P = 0.001) than placebo. Although the risk of abdominal pain was higher in nintedanib than placebo, the difference did not reach statistical significance (10.5% vs 4.9%; OR = 2.19; 95% CI = 1.00–4.77; P = 0.05), Whereas, patients treated with nintedanib were less likely to have a cough (12.1% vs 15.6%; OR = 0.73; 95% CI = 0.56–0.96; P = 0.02), and dyspnea (8.9% vs 12.5%; OR = 0.70; 95% CI = 0.53–0.94; P = 0.02) (Fig 3 and S1 Fig).

### Sensitivity analysis

Studies were removed one at a time and it was determined that no individual trial had a significant impact on the overall pooled results for all adverse events outcomes. The magnitude and direction of association were consistent with the results when all studies were pooled together (Table 3).

**Table 3:** Leave-one-out sensitivity analyses for adverse events.

	Statistics with study removed			
Study name	OR	Lower limit	Upper limit	P-value
**Any adverse events**				
INBUILD	2.35	1.57	3.52	<0.001
INPULSIS-1	2.18	1.49	3.19	<0.001
INPULSIS-2	2.64	1.78	3.92	<0.001
NCT01979952	2.37	1.67	3.34	<0.001
SENSCIS	2.39	1.67	3.41	<0.001
TOMORROW	2.48	1.74	3.53	<0.001
**Severe adverse events**				
INBUILD	1.26	0.96	1.66	0.093
INPULSIS-1	1.02	0.76	1.38	0.885
INPULSIS-2	1.17	0.79	1.73	0.436
SENSCIS	1.04	0.75	1.44	0.809
TOMORROW	1.16	0.82	1.63	0.402
**Serious adverse events**				
INBUILD	1.02	0.83	1.25	0.873
INPULSIS-1	0.95	0.79	1.15	0.615
INPULSIS-2	1.04	0.86	1.27	0.693
NCT01979952	1.01	0.85	1.20	0.911
SENSCIS	0.97	0.80	1.17	0.721
TOMORROW	1.01	0.85	1.21	0.901
**Adverse events leading to treatment discontinuation**				
INBUILD	1.62	1.19	2.21	0.002
INPULSIS-1	1.63	1.21	2.21	0.001
INPULSIS-2	1.95	1.50	2.53	<0.001
SENSCIS	1.67	1.21	2.31	0.002
TOMORROW	1.82	1.37	2.42	<0.001
**Fatal adverse events**				
INBUILD	0.68	0.33	1.37	0.279
INPULSIS-1	0.64	0.32	1.26	0.194
INPULSIS-2	0.62	0.29	1.31	0.211
SENSCIS	0.63	0.36	1.10	0.101
TOMORROW	0.77	0.52	1.15	0.197

For the most frequent adverse events, no individual study had a significant impact on the magnitude and direction of association for diarrhea, nausea, vomiting or weight loss. As for cough, the pooled results became insignificance when the INBUILD, INPULSIS-2, NCT01979952, SENSCIS or TOMORROW studies were removed one at a time. As for nasopharyngitis, the pooled results changed from insignificant to significant if the INBUILD study was removed (S1 Table).

### Quality assessment

**Fig 4:**
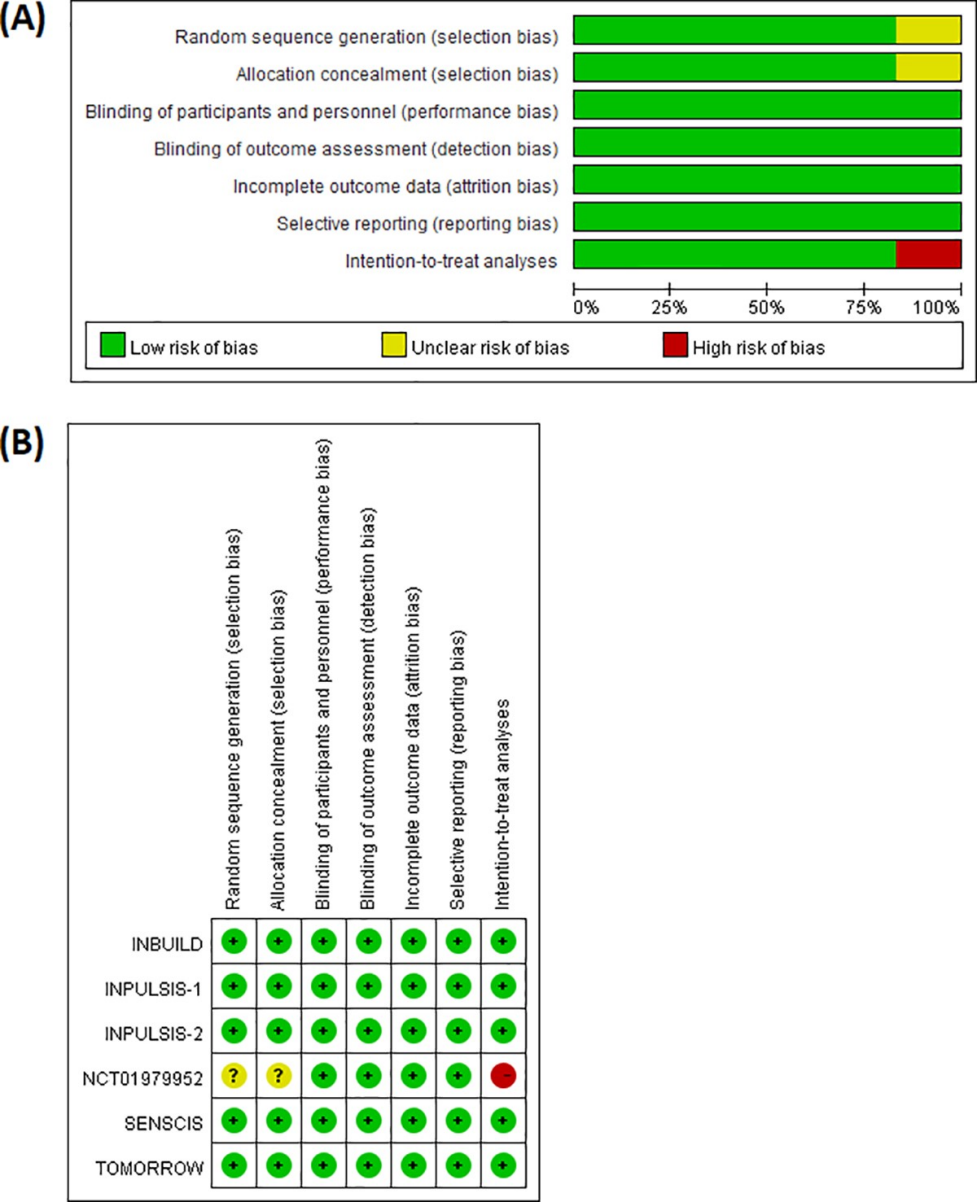
Quality assessment of included studies. (A) Risk of bias summary for the included studies, (B) Risk of bias for each individual study.

Fig 4 summarizes the overall quality of the included studies and the risk of bias for individual trials included in the meta-analysis. All studies were double-blind and had a low risk of attrition and reporting bias (Fig 4A). One unpublished trial had unclear randomization procedures and did not analyze the data in the intention-to-treat approach (Fig 4B) [25].

## Discussion

The pooled analysis of safety data from the 6 clinical trials for patients with IPF and fibrotic-ILDs, showed that adverse events were more frequent in patients treated with nintedanib compared with the placebo. There was a higher likelihood of having adverse events leading to treatment discontinuation, but a lower likelihood of having fatal adverse events.

The most common adverse event associated with nintedanib use compared with the placebo was diarrhea. The underlying cause of diarrhea development remains unclear, but it may be due to direct irritation of the gastrointestinal (GI) tract due to high concentrations of the drug [26]. For most patients with an adverse event of diarrhea, first onset occurred within the first 3 months after the administration of nintedanib [27]. In a subgroup analysis of the INPULSIS trial [28], Japanese patients suffered from diarrhea more frequently compared with the overall population (75.0% vs. 62.4%). A retrospective study showed that low body mass index (BMI; <21.6) was a risk factor for diarrhea [29]. If diarrhea develops, adequate hydration and anti-diarrheal medication (*e*.*g*. loperamide) are suggested [30]. Treatment interruption and dose reduction may only be necessary if diarrhea persists despite treatment. Most patients with diarrhea were able to control it with treatment, and only 4.5% (0.6% in the placebo) needed permanent discontinuation of nintedanib in the pooled data from the TOMORROW and INPULSIS trials [8].

The risk of diarrhea was consistently higher in the nintedanib group compared with the placebo group. However, in the INBUILD and SENSCIS studies, the prevalence of diarrhea remained higher than in the TOMORROW and INPULSIS trials in both the nintedanib (71.0% vs. 61.5%) and placebo groups (27.5% vs. 17.9%). In addition, the proportion of patients with nausea and vomiting in the INBUILD and SENSCIS trials were also higher than in the other trials. This may be because the INBUILD and SENSCIS trials included patients with autoimmune-related ILD. All patients in the SENSCIS trial had SSc, and 25.6% of patients in the INBUILD trial had an autoimmune disease (rheumatoid arthritis 13.4%, SSc 5.9%, mixed connective tissue disease (MCTD) 2.9%, other 3.5%). Patients with an autoimmune disease had more systemic involvement and a higher prevalence of diarrhea (50% in SSc patients and 8% in MCTD patients) compared with IPF before the administration of nintedanib [31, 32]. Approximately 90% of patients with SSc had some degree of GI involvement [33], and the use of nintedanib may have to be more carefully considered/monitored in these patients.

Nausea, vomiting, weight loss, and decreased appetite were other adverse events that occurred significantly more frequently in the nintedanib group compared with the placebo group. A poor performance status (ECOG PS 2–4), and a low BMI were risk factors for nausea [29]. Most of these adverse events were of a mild to moderate intensity and less frequently led to the discontinuation of nintedanib compared with diarrhea [6]. Elevation of hepatic enzymes also occurred more frequently in patients treated with nintedanib compared with the placebo group. One study pooling results from the TOMORROW, INPULSIS and NCT01979952 trials reported a higher rate of liver enzyme elevation in the nintedanib group compared with the placebo group among IPF patients [19]. Similar results were also found in the INBUILD trial. Patients with a progressive fibrotic phenotype of ILD were more likely to have elevated ALT and AST after treatment with nintedanib (13.0% vs. 3.6% for ALT and 11.4% vs. 3.6% for AST). Elevation of hepatic enzymes may be an early presentation of hepatotoxicity and asymptomatic, periodic monitoring prior to and during treatment with nintedanib should be conducted. Elevated hepatic enzymes is almost always reversible with dose reduction or treatment interruption [30].

Although the placebo is a dummy medication without pharmacological effects, >90% of patients in the placebo group suffered from some kind of adverse event across all 6 included trials. One possible explanation for this is the nocebo effect, a phenomenon where negative effects are attributed to the placebo [34, 35]. On the other hand, clinical characteristics of disease *per se* may also explain the higher proportion of adverse events in the placebo groups. For example, a certain degree of GI involvement occurs in autoimmune-related ILD [33]. Furthermore, in our pooled results, respiratory-related adverse events were more frequent in the placebo group, such as a cough, bronchitis, dyspnea, nasopharyngitis, and upper respiratory tract infection. These may be related to the high prevalence of respiratory symptoms in IPF patients, such as dyspnea (54–98%) or cough (59–100%) [36]. A study analyzing the serious adverse events in the placebo arms of 6 randomized clinical trials for interferon-γ 1b or pirfenidone, found that respiratory-related conditions, infections and infestations were the most frequently reported serious adverse events in IPF patients [37]. These results imply that respiratory-related adverse events are more likely to be related to the progression of IPF or PF-ILD itself than to the medication, and may evaluate as trial outcome parameters rather than adverse events. Discontinuation of nintedanib due to such adverse events may not be helpful.

The exacerbation rate of ILD was lower in nintedanib group compared with in placebo group, although presented in different definition across these studies as following (not mentioned in SENSCIS): 7.8% vs 9.8% (INBUILD, acute exacerbation of ILD or death at 52 weak); 6.1% vs 5.4%, 3.6% vs 9.6% (INPULSIS-1&2, proportion of patients with at least one investigator reported acute exacerbation); 1.8% vs 1.8% (NCT01979952, percentage of subjects experienced first acute IPF exacerbations between 0 to 6 months); 2.4 vs 15.7 (TOMORROW, per 100 patient-years). The hospitalization rate was only described in NCT01979952 (0.0% vs 7.0%, percentage of subjects hospitalized due to respiratory problems between 0 to 6 months) and TOMORROW (27.1% vs 25.9%, adverse events requiring hospitalization) studies. In other studies, only the serious adverse events, which including adverse events that resulted in hospitalization, were reported (Table 2).

A strength of the current analysis was the fact that it is the first meta-analysis to include studies of fibrotic-ILD patients as well as IPF patients. It provides a more comprehensive view of the safety profile of nintedanib in ILD patients. We only included high quality RCTs and compared with a placebo to avoid possible confounding factors. A limitation of this analysis is the fact that the included trials excluded patients with severely impaired pulmonary function, certain comorbidities, or current medication. This may lead to the results having a different prevalence of adverse events after treatment with nintedanib compared with patients in the real world.

## Conclusions

Compared to a placebo, nintedanib is associated with a higher risk of adverse events, especially diarrhea, nausea and weight loss, but it was also associated with a lower risk of cough and dyspnea in IPF and fibrotic-ILD patients. Nintedanib had similar risk of adverse events in fibrotic-ILD compared with in IPF patients, but higher prevalence of diarrhea, nausea or vomiting in fibrotic-ILD patients suggested careful management of these adverse events if use nintedanib in fibrotic-ILD patients.

## Supporting information

S1 Checklist PRISMA 2009 checklist. (DOC)

S1 Fig Forest plots for association between the other adverse events and use of nintedanib. (TIFF)

S1 Table Leave-one-out sensitivity analyses for most frequent adverse events. (DOCX)
