# Supplementary figures and images for: The safety of nintedanib for the treatment of interstitial lung disease: A systematic review and meta-analysis of randomized controlled trials
Source: PLoS One. 2021 May 14;16(5):e0251636. doi: 10.1371/journal.pone.0251636 (PMC8121296; doi:10.1371/journal.pone.0251636)

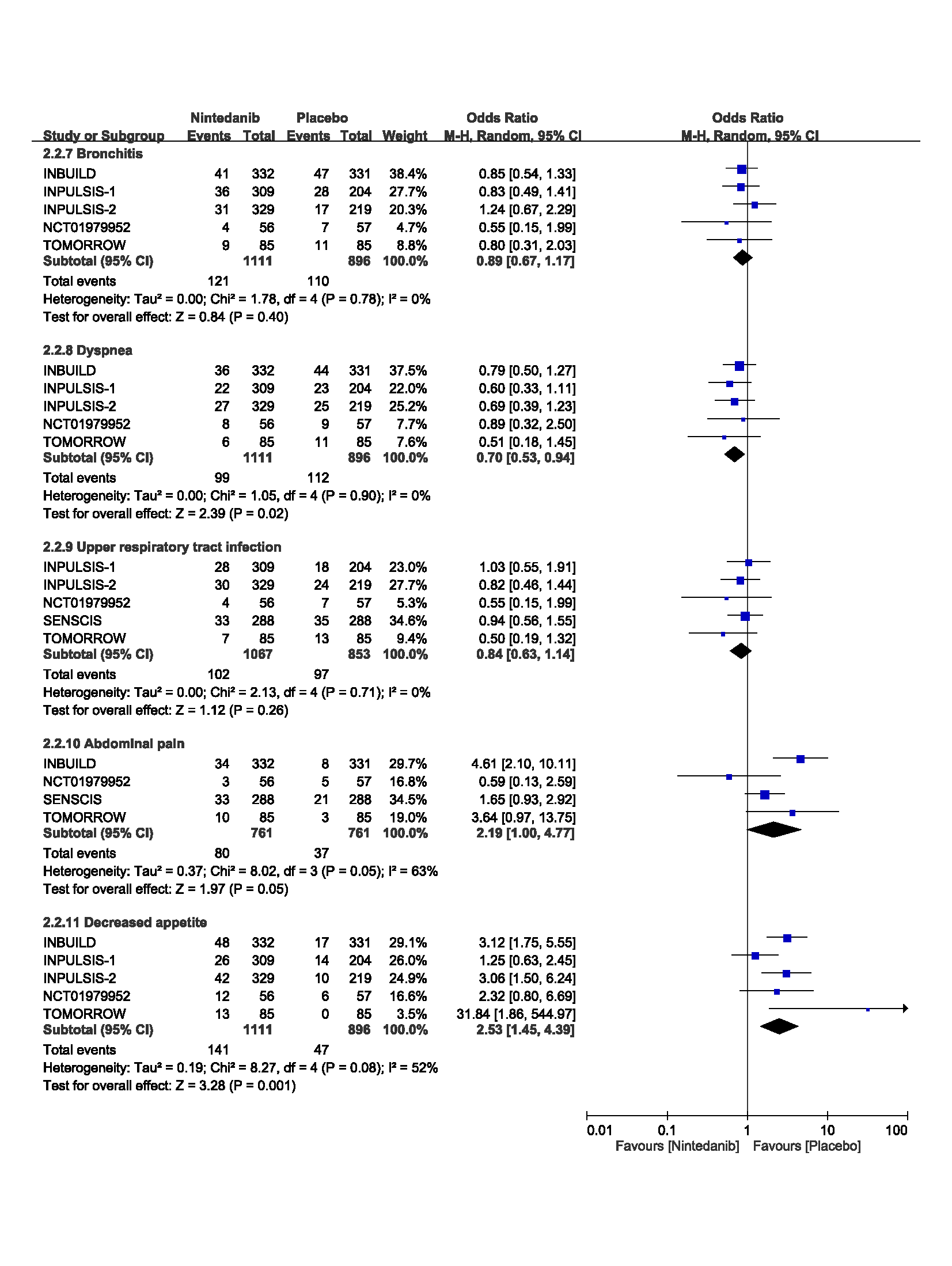

Supplement: S1 Fig — (TIFF) [file pone.0251636.s002.tiff]
