# Supplementary material for: The safety of nintedanib for the treatment of interstitial lung disease: A systematic review and meta-analysis of randomized controlled trials
Source: PLoS One. 2021 May 14;16(5):e0251636. doi: 10.1371/journal.pone.0251636 (PMC8121296; doi:10.1371/journal.pone.0251636)
Supplement: S1 Table — (DOCX) [file pone.0251636.s003.docx]

| **S1 Table. Leave-one-out sensitivity analyses for most frequent adverse events** | | | | |
| --- | --- | --- | --- | --- |
|  | **Statistics with study removed** | | | |
| **Study name** | **OR** | **Lower limit** | **Upper limit** | **P-value** |
| **Diarrhea** |  |  |  |  |
| INBUILD | 5.68 | 3.75 | 8.62 | <0.001 |
| INPULSIS-1 | 5.64 | 3.80 | 8.35 | <0.001 |
| INPULSIS-2 | 5.53 | 3.76 | 8.12 | <0.001 |
| NCT01979952 | 6.88 | 5.73 | 8.27 | <0.001 |
| SENSCIS | 5.64 | 3.75 | 8.48 | <0.001 |
| TOMORROW | 5.79 | 4.05 | 8.28 | <0.001 |
| **Nausea** |  |  |  |  |
| INBUILD | 2.78 | 1.59 | 4.86 | <0.001 |
| INPULSIS-1 | 2.74 | 1.65 | 4.54 | <0.001 |
| INPULSIS-2 | 2.74 | 1.63 | 4.60 | <0.001 |
| NCT01979952 | 3.68 | 2.91 | 4.67 | <0.001 |
| SENSCIS | 2.96 | 1.66 | 5.29 | <0.001 |
| TOMORROW | 2.99 | 1.81 | 4.93 | <0.001 |
| **Vomiting** |  |  |  |  |
| INBUILD | 2.97 | 1.83 | 4.83 | <0.001 |
| INPULSIS-1 | 2.95 | 2.06 | 4.22 | <0.001 |
| INPULSIS-2 | 3.16 | 1.96 | 5.10 | <0.001 |
| NCT01979952 | 3.54 | 2.61 | 4.79 | <0.001 |
| SENSCIS | 3.36 | 1.98 | 5.71 | <0.001 |
| TOMORROW | 3.24 | 2.07 | 5.07 | <0.001 |
| **Cough** |  |  |  |  |
| INBUILD | 0.73 | 0.51 | 1.03 | 0.075 |
| INPULSIS-1 | 0.66 | 0.51 | 0.85 | 0.001 |
| INPULSIS-2 | 0.71 | 0.51 | 1.00 | 0.052 |
| NCT01979952 | 0.75 | 0.56 | 1.01 | 0.060 |
| SENSCIS | 0.77 | 0.56 | 1.07 | 0.115 |
| TOMORROW | 0.77 | 0.59 | 1.01 | 0.055 |
| **Nasopharyngitis** |  |  |  |  |
| INBUILD | 0.74 | 0.57 | 0.96 | 0.025 |
| INPULSIS-1 | 0.85 | 0.66 | 1.09 | 0.197 |
| INPULSIS-2 | 0.79 | 0.61 | 1.02 | 0.071 |
| NCT01979952 | 0.83 | 0.66 | 1.05 | 0.122 |
| SENSCIS | 0.86 | 0.66 | 1.12 | 0.258 |
| TOMORROW | 0.84 | 0.66 | 1.06 | 0.136 |
| **Weight loss** |  |  |  |  |
| INBUILD | 3.29 | 1.42 | 7.63 | 0.006 |
| INPULSIS-1 | 4.14 | 2.59 | 6.61 | <0.001 |
| INPULSIS-2 | 2.73 | 1.55 | 4.79 | <0.001 |
| NCT01979952 | 3.32 | 1.55 | 7.11 | 0.002 |
| SENSCIS | 3.63 | 1.49 | 8.86 | 0.005 |
